# Prevalence and risk factors of intimate partner violence during the COVID-19 pandemic: Results from the population-based study DREAM_CORONA_

**DOI:** 10.1371/journal.pone.0306103

**Published:** 2024-06-27

**Authors:** Amera Mojahed, Judith T. Mack, Andreas Staudt, Victoria Weise, Lakshmi Shiva, Prabha Chandra, Susan Garthus-Niegel

**Affiliations:** 1 Faculty of Medicine Carl Gustav Carus, Department of Psychotherapy and Psychosomatic Medicine, Technical University of Dresden, Dresden, Germany; 2 Department of Methods in Community Medicine, Institute of Community Medicine, University Medicine Greifswald, Greifswald, Germany; 3 Aasare Neuropsychiatric Centre, Bangalore, India; 4 National Institute of Mental Health and Neurosciences, Bangalore, India; 5 Faculty of Medicine, Institute for Systems Medicine, Medical School Hamburg, Hamburg, Germany; 6 Department of Childhood and Families, Norwegian Institute of Public Health, Oslo, Norway; Caleb University, NIGERIA

## Abstract

**Objectives:**

This study examines the 12-month prevalence rates of intimate partner violence (IPV) victimization, including psychological, physical, and sexual forms, in women and men. It also aims to identify changes in IPV victimization during the COVID-19 pandemic and to explore factors associated with the occurrence of any IPV victimization during this period.

**Methods:**

Data from the DREAM_CORONA_ study in Germany collected from May 2020 to February 2021 included 737 participants, i.e., (expectant) mothers (64%) and fathers (36%). The Revised Conflict Tactics Scale (CTS2S) short form was used to assess the 12-month IPV victimization. Prevalence of IPV victimization as well as changes in IPV victimization during the pandemic were analyzed descriptively, with results stratified by sex. Multiple logistic regression was employed to identify risk factors for IPV.

**Results:**

Psychological IPV was found to be the most prevalent form of violence, with the occurrence of any psychological IPV affecting 48.5% of women and 39.4% of men, while 2.6% of women and 3.3% of men reported the occurrence of any physical IPV victimization, and 2.8% of women and 1.5% of men reported the occurrence of any sexual IPV victimization. Of those who experienced the occurrence of any IPV in the last 12 months, 89.7% of women and 89.8% of men were victimized by one single act of violence. The majority of affected participants reported no change in psychological and physical IPV victimization during the pandemic. Nevertheless, for certain IPV behaviors on the psychological and physical IPV victimization subscales, both affected women and men also reported higher frequencies during the COVID-19 pandemic. Multiple logistic regression revealed that higher levels of relationship satisfaction were negatively associated with the occurrence of any IPV victimization for women and men, whereas greater levels of own anger-hostility symptoms were positively associated with the occurrence of any IPV victimization.

**Conclusions:**

Psychological IPV was present in almost every second (expectant) couple. The majority of affected women and men reported no change in their psychological and physical IPV victimization, suggesting that they continued to experience IPV during the pandemic. This underlines the importance of promoting healthier relationship dynamics, coping strategies, and emotional well-being to reduce the risk of IPV, even in times of crisis. Our study sheds light on the early stages of the pandemic and highlights the ongoing need for research into the temporal dynamics of IPV.

## Introduction

### Prevalence rates of IPV victimization

Intimate partner violence (IPV), also known as domestic violence, consists of a pattern of assaultive and coercive behaviors, including physical, sexual, and psychological attacks, as well as economic coercion, by a current or former intimate partner. It can occur within heterosexual or same-sex relationships and does not require having sexual relations [1,2]. According to the World Health Organization’s most recent summary of prevalence estimates of violence against women [2], one in four women worldwide is estimated to be subjected to physical and/or sexual violence by an intimate partner at least once in her lifetime.

In a recent German national study on IPV victimization, lifetime psychological IPV was the most common form, reported by 53.6% of women and 48.0% of men. Other forms ranged from 15.2% for lifetime physical IPV to 18.6% for lifetime sexual IPV for women. For men, lifetime physical and sexual IPV was reported by 10.8% and 5.5% respectively [3]. While this study primarily examined the lifetime prevalence of IPV in Germany, knowledge gaps remain. In particular, there is still a lack of information on perinatal women and men in Germany [4]. Examining prevalence rates, particularly within the perinatal period, which includes the year before conception, the time throughout pregnancy and up to one [5] or two years after birth [6], would be particularly informative as it covers critical periods in the family life cycle when stressors and dynamics may be heightened. Perinatal IPV can significantly affect the health and well-being of individuals before, during, and after pregnancy. For (expectant) women, the consequences include various physical health issues such as the increased risk of prenatal hemorrhage [7], preterm birth [8], and sexually transmitted infections (STIs) [9]. In addition, mental health effects may include depression [10], anxiety [11], post-traumatic stress disorder (PTSD) [10], suicidal thoughts, and the committing of suicide [12].

### Changes in IPV victimization during the COVID-19 pandemic

(Perinatal) IPV exists within a broader context that includes political, social, and environmental influences [13]. As such, previous empirical literature showed that the experience of IPV is impacted by significant societal events and times of crisis such as pandemics and natural disasters [14,15]. For example, during the 2014–⁠2016 Ebola epidemic [14] and in the 2008 post-earthquake period in Sichuan, China [15] estimates of IPV increased compared to the years preceding those events. These events contributed to an increased economic strain on the household, restrictions on movement, as well as the disruption of ongoing social services, which were in turn main drivers for increased IPV. Similarly, emerging research has shown that the COVID-19 pandemic might have predisposed some partners to increased conflict and IPV [16–18].

According to previous studies, trends or changes in IPV victimization have been measured in order to develop a more comprehensive and inclusive understanding of IPV in different populations and during a societal crisis such as the COVID-19 pandemic [18]. In 2020, a German study reported psychological IPV victimization against women had increased by about 77% and against men by about 78% [19]. An increase in physical IPV victimization of almost 15% for women and 21% for men was also observed. In addition, the study reported a 3% increase in sexual IPV victimization against women but none of the men reported experiencing increased sexual IPV victimization [19]. In 2021, another German study found no significant changes of reported 12-month IPV rates (2016 versus 2021) [20]. To the best of our knowledge, no previous survey data have reported a decline in IPV victimization in Germany. Upon these existing yet conflicting findings, i.e., an increase vs. no change in victimization, in current literature and the lack of research on the changes in IPV victimization during the pandemic in perinatal female and male populations in Germany, there seems to be a need to address this research gap.

### Possible factors associated with IPV victimization

Previous studies have identified several factors associated with (perinatal) IPV. Mental disorders, such as depression and anxiety, have been found to be associated with physical and psychological IPV victimization and perpetration in the general female population [4] as well as any occurrence of IPV victimization in perinatal female populations [10,21]. This finding is consistent with another study that showed that pregnant women who were physically and sexually abused experienced significantly elevated levels of negative emotions such as depression and anxiety, along with increased distress and loneliness [22]. Howard’s 2013 systematic review suggests that experiencing IPV during pregnancy may increase the burden of postpartum mental disorders [21], underscoring the importance of addressing IPV as a public health concern. Furthermore, individual longitudinal studies, which were included in the review [21], suggest a 3- to 5-fold increased likelihood of women experiencing IPV during or up to one year after pregnancy if they have probable depression during the prenatal period. Although causality cannot be definitively established, these findings suggest a bidirectional relationship between exposure to IPV and probable depression in both the prenatal and postnatal periods. While the mental health effects of IPV victimization during the perinatal period may stem from the combined impact of stress, hormonal changes, and lack of support in (expectant) women [23], it is theoretically plausible that e.g., depression symptoms may increase the risk of IPV victimization through a reduced cognitive and affective capacity by which to recognize threat [24,25], thereby complicating decision-making and responses to danger cues.

In the context of the COVID-19 pandemic, mental disorders could have been aggravated by the increase in situational stressors associated with the COVID-19 pandemic, such as economic stress, social isolation, job loss, or illness [17,26], which could have further increased the risk of violent episodes occurrence, as recent research has shown [27]. A study reported that women’s experiences of physical, sexual, and psychological IPV during the early days of the pandemic were all positively associated with more symptoms of depression [28]. With regard to male populations, research has focused more on mental disorders associated with male perpetration of IPV rather than victimization [4]. As a result, there is a significant gap in our understanding of the mental disorders associated with male victimization during the perinatal period, globally and in Germany.

Anger-hostility symptoms have been extensively examined in studies investigating the risk of perpetration of IPV [29]. Previous research on IPV victimization only suggests a pattern where individuals who later become aggressors often exhibit anger, hostility, and internalization of negative emotions as a result of prior victimization experiences [29,30]. However, a notable gap remains in research of the association of one’s own anger-hostility symptoms with IPV victimization (independent of one’s own perpetration). Regarding the scope of the COVID-19 pandemic, previous studies found that the initial and long-term stress caused by this pandemic could have contributed to emotional dysregulation problems, such as hostility symptoms [18,31,32]. In Australia, a study found a 42% increase in reported incidents of IPV occurring in relationships that had not previously been abusive, due to situational stressors related to the COVID-19 pandemic and emotional dysregulation [16]. For relationships with a history of abusive behaviors, the added stressors of confined living situations for cohabiting partners during the pandemic, where privacy and personal space had been lacking, could have escalated the frequency and severity of abuse [33,34].

While coping encompasses strategies in a broad sense for managing life stressors, dyadic coping involves both individuals in a couple and focuses on the dynamic interplay between one partner’s stress *cues* and the other’s coping responses [35]. Dyadic coping serves a dual purpose: it reduces stress for both partners and improves the quality of the relationship. When one partner faces a significant stressor, or when both members of a couple face a common challenge, the use of dyadic coping strategies is expected to reduce distress [36]. Recognized as essential to relationship quality and well-being, positive dyadic coping cultivates mutual trust, respect, commitment, and a sense that the relationship provides comfort and support [37]. Conversely, negative dyadic coping includes behaviors that could constitute open disinterest or sarcasm about a partner’s stress, using inauthentic coping strategies, responding reluctantly to stress cues, or doubting the actual need for support [36]. Despite the acknowledged importance of dyadic coping in relationship quality and overall well-being [37], there remains a gap in research regarding the potential association between negative dyadic coping and perinatal IPV victimization. In addition, recent dyadic research has found that both lower relationship satisfaction is associated with IPV victimization in the general population [38,39]. However, there remains a lack in our understanding of the individual perspectives of perinatal women and men.

Social isolation, characterized by a lack of meaningful relationships and support networks, has emerged as a significant factor contributing to IPV victimization [33], with the COVID-19 pandemic measures potentially exacerbating this issue. While the existing literature highlights the positive association between social isolation and IPV predominantly in (perinatal) women [33], there is a notable gap in research regarding social isolation and IPV in (perinatal) men. Finally, known risk factors that relate to socio-demographic characteristics such as young age, lower and higher education, being pregnant, having more than one child, and living in a large household with, for example, more than one child under the age of five, have been associated with (perinatal) IPV victimization in women [4,40–42]. This diverse array of risk factors suggests a complex interplay of socio-demographic, life stage, and family elements that contribute to women’s vulnerability during the perinatal period. Similar to women, potential risk factors for men could also include these mentioned factors. Yet research on perinatal men in Germany remains sparse.

Therefore, our study replicates the existing research, fills in gaps, and extends it to a pandemic context, i.e., examines whether the same risk factors apply during a pandemic and adds information on risk factors of perinatal IPV victimization not only in women, but also in men in Germany. Given these considerations, the current study aimed to: 1) explore the 12-month prevalence of the occurrence of psychological, physical, and sexual IPV victimization as well as the occurrence of any IPV victimization in (expectant) women and men; to 2) explore any possible changes in the experienced IPV behaviors during the COVID-19 pandemic in affected (expectant) women and men; and to 3) explore potential risk factors during the COVID-19 pandemic that could be associated with the occurrence of any IPV victimization in (expectant) women and men. Based on most recent findings, we generated the following hypotheses:

- The 12-month prevalence rates of perinatal IPV victimization will be close to those observed in the general population in Germany.
- The change in IPV victimization analysis will reveal an expected increase or persistence during the pandemic.
- The multiple logistic models will demonstrate young age, being pregnant, large household size, higher psychological symptoms (depression or anger-hostility symptoms), negative dyadic coping, lower relationship satisfaction, and/or lower social support as potential risk factors of the occurrence of any IPV victimization. In addition, having more or less than 10 years of education and being expectant first-time parent or already having one or more children will be included and explored.

## Materials and methods

### Study design and participants

The prospective cohort study "Dresden Study on Parenting, Work, and Mental Health" (referred to as **DR**esdner Studie zu **E**lternschaft, **A**rbeit und **M**entaler Gesundheit, **DREAM**), explores the interplay between parental engagement in employment, distribution of roles within families, stress factors, and their impact on the family mental and somatic health [43]. The recruitment phase for the study employed a population-based convenience sampling approach and spanned from June 2017 until the end of 2020. The study’s community sample comprised women who were pregnant during the recruitment phase and their partners, all of whom were residing in Dresden, Germany, or its surrounding regions at that time.

As part of the DREAM study, participants were given the option to complete questionnaires related to various aspects of their mental and physical health either through paper forms or online. The DREAM study encompasses six measurement points: T1 during pregnancy, T2 at 8 weeks postpartum, T3 at 14 months postpartum, T4 at 2 years postpartum, T5 at 3 years postpartum, and T6 at 4.5 years postpartum. Additional details regarding the DREAM study are elaborated upon in the corresponding study protocol [43].

Data on IPV and other relevant variables were derived from the population-based longitudinal sub-study DREAM_CORONA_. In response to the outbreak of the pandemic, the DREAM_CORONA_ study was established as an addition to the regular assessments of DREAM. The DREAM_CORONA_ sub-study investigates experiences of (expectant) parents during the COVID-19 pandemic (e.g., social isolation, school and daycare closures, working from home) and its impact on family health, role distributions, and relationships.

The DREAM_CORONA_ study population participated originally in the DREAM study. Those who completed at least the first measurement point in the main DREAM study by May 2020 and were registered as online participants were invited to the DREAM_CORONA_ study. Pandemic-related restrictions hindered sending out study material, particularly affecting the delivery of paper-pencil versions. Additionally, invitations were extended exclusively to participants with singleton pregnancies; parents of twins or multiples did not receive an invitation due to feasibility constraints.

**Fig 1:**
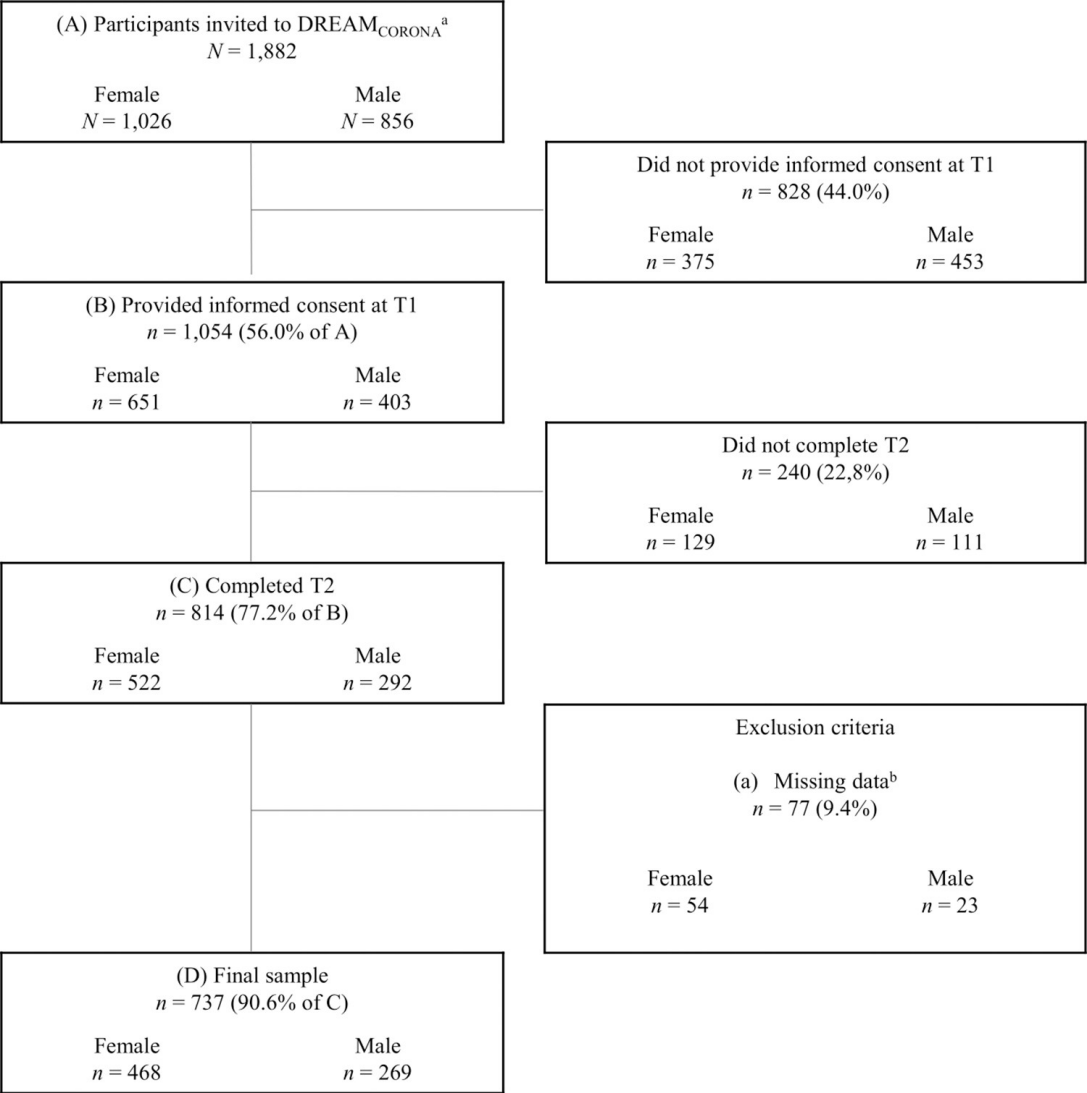
Flow chart of final sample.

The DREAM_CORONA_ sub-study comprises two measurement points. Invitations for the T1 online survey were sent out via mail on May 12, 2020, followed by two reminders after three and six days. Invitations for the T2 online follow-up survey were sent from October 20, 2020, using the same reminder system. Between May 12 and October 1, 2020, a total of 1,882 individuals were invited to partake in the study. Out of this group, 1,054 individuals gave written consent to participate in DREAM_CORONA_, resulting in a response rate of 56%, and of which 814 participants took part in the follow-up (T2; Fig 1).

**Fig 2:**
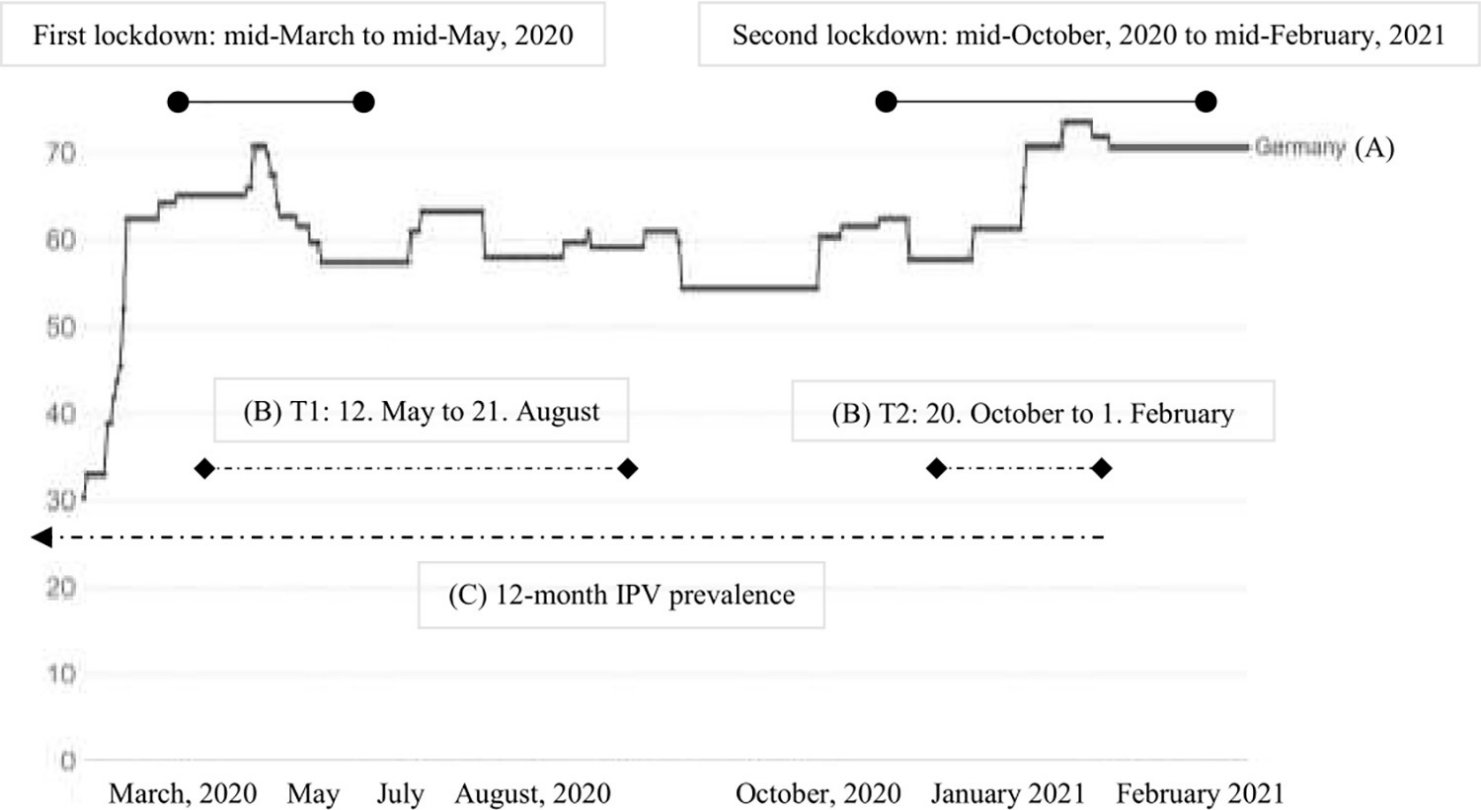
Timeline of the DREAM_CORONA_ study, the 12-month IPV victimization prevalence, and COVID-19 lockdowns in Germany.

Further, as this study investigates victimization prevalence and risk factors for IPV, we included only participants who completed the IPV questionnaire at T2 of the DREAM_CORONA_ study (*N* = 737). This resulted in a sample of 468 women and 269 men (Fig 1). An overview of the data collection points, the 12-month IPV prevalence, as well as the lockdowns in Germany is illustrated in Fig 2 based on the containment and health index. This index is a composite measure based on thirteen policy response indicators including school and workplace closures, travel bans, testing policy, contact tracing, face coverings, and vaccine policy rescaled to a value from 0 to 100 (100 = strictest) [44]. Fig 2 is intended to be a visual aid that allows readers to draw a connection between the stringency of the containment measures imposed during the pandemic and the 12-month IPV prevalence.

### Measures

#### Independent variables

*Socio-demographic characteristics*. Variables included in our study were self-reported sex, age, education, pregnancy status, being expectant first-time parent, household size, sex of partner, and cohabitation [45]. Education was dichotomized into ≤ 10 years of school education, and ˃ 10 years of school education. Being expectant first-time parent was assessed in terms of ‘yes, being expectant first-time parent’ (0) or ‘no, already having one or more children’ (1) to date. Cohabitation was assessed with an item asking the participants ‘Do you live with your partner?’ and the response options ‘yes, all the time’ (1), ‘yes, but not all the time (e.g., only at weekends)’ (2), or ‘no’ (3). Education was the only variable derived from the baseline assessment of the main DREAM study, whereas all other independent variables were taken from T1 of the DREAM_CORONA_ study.

*Relationship satisfaction*. The German short version of the partnership questionnaire (PFB-K) was used to measure self-reported relationship satisfaction [46]. It consists of three subscales: Quarreling, tenderness, togetherness/communication, with three items respectively. Response categories range from ‘never/very rare’ (1), ‘rare’ (2), ‘often’ (3), to ‘very often’ (4). The total score for the whole scale varies from a minimum of 9 to a maximum of 36. A higher score implicates greater relationship satisfaction.

*Dyadic coping*. The 37-item Dyadic Coping Inventory (DCI) [47] assesses one’s own stress communication ‘What do I do when I am stressed?’ and that enacted by the partner ‘What does my partner do when he/she is stressed?’ Furthermore, positive and negative forms of supportive dyadic coping as perceived by the self ‘What do I do when my partner is stressed?’ and in the partner ‘What does my partner do when I am stressed?’ are assessed. Also, each partner’s view of how they cope as a couple (common dyadic coping) is evaluated ‘What do we do when we are stressed as a couple?’. Items are answered on 5-point scales ranging from 1 ‘never’ to 5 ‘very often’. In this study we used the sum score of the DCI, i.e., the sum of items 1 through 35 after reverse coding negatively keyed items (7, 10, 11, 15, 22, 25, 26, and 27). Items 36 and 37 are evaluation items, e.g., ‘I am satisfied with the support I receive from my partner and the way we deal with stress together’, and not included in the sum score. The total score for the whole scale varies from a minimum of 35 to a maximum of 175. Higher values indicate better or more positive coping.

*Psychological symptoms*. Symptoms of depression during the past week were measured by the German Edinburgh Postnatal Depression Scale (EPDS). The EPDS is a self-rating 10-item scale. Each answer is scored on a scale from 0 to 3, and the total score for the whole scale varies from a minimum of 0 to a maximum of 30 [48–52]. An example item is ‘I was sad and miserable.’ Higher values indicate higher levels of depression symptoms.

Other psychological symptoms related to anger-hostility were assessed by the Symptom Checklist (SCL-90-R). Each of the 6 items was rated on a 5-point Likert scale, ranging from 0 ‘not at all’ to 4 ‘extremely’, assessing severity of the symptoms in the last seven days [53–57]. An example item is ‘How much did you suffer from feeling easily irritated or upset?’. The total score (0–24) was used with higher values indicating higher levels of anger-hostility symptoms.

*Social support*. Perceived social support was assessed by the short form of the Social Support Questionnaire (‘Fragebogen zur Sozialen Unterstützung’, F-SozU-14 [58]. The F-SozU K-14 contains 14 items answered on a 5-point scale ranging from 1 ‘does not apply’ to 5 ‘does exactly apply’. The total score for the whole scale varies from a minimum of 14 to a maximum of 70. The items refer to practical and emotional support, social integration, satisfaction with social support, and availability of confidants. Example items are ‘I experience a lot of understanding and security from others.’ and ‘I know a very close person whose help I can always count on.’ The sum score indicates participants’ overall levels of perceived social support.

#### Dependent variables

*IPV in the last 12 months*. The T2 survey in the DREAM_CORONA_ study included questions that assessed both the nature and extent of IPV. IPV victimization was assessed with the *short form of the Revised Conflict Tactics Scale* (CTS2S); a self-report measure of tactics used during relationship conflicts of dating, cohabiting, or married couples [59].

The 20 items of the CTS2S are divided into five subscales (i.e., two items per subscale): negotiation, psychological aggression, physical assault, sexual coercion, and injury. The psychological aggression, physical assault, and sexual coercion subscale measure reprehensible or violent behaviors or acts that have been committed by a current partner or ex-partner against the respondent (victimization measure), as well as the violent behaviors that have been perpetrated by the respondent her-/himself (perpetration measure).

In this study, only the victimization measure scores of the 3 self-reported subscales for the 3 forms of IPV (i.e., psychological aggression, physical assault, and sexual coercion) were used for the prevalence measures. Each of the items represented an individual behavior within the victimization measure used for the prevalence measures. They included, for example, ‘My partner insulted or swore or shouted or yelled at me’ (psychological aggression), ‘My partner punched or kicked or beat me up’ (physical assault), and ‘My partner used force (like hitting, holding down, or using a weapon) to make me have sex’ (sexual coercion).

Participants in the current study were asked to indicate the occurrence of victimization by each of the violent behaviors in their relationship with their current partner by indicating the frequency of occurrence of conflict tactics ranging from 1 ‘once in the past year’ to 6 ‘more than 20 times in the past year’, with 7 and 8 indicating ‘not in the past year, but it happened before’ and ‘this has never happened’, respectively. According to Straus (2004), the CTS2S can be used not only as a frequency measure, but also as a prevalence measure of violence victimization [59]. Thus, in this study we dichotomized the answers, ‘yes’ (1) and ‘no’ (0), by indicating if the behaviors had occurred or not in each of the psychological aggression (i.e., one or more of either of the two items “occurrence of any psychological IPV victimization”), physical assault (i.e., one or more of either of the two items “occurrence of any physical IPV victimization”), and sexual coercion subscales (i.e., one or more of either of the two items “occurrence of any sexual IPV victimization”), as well as any IPV behavior (i.e., across the six items “occurrence of any IPV victimization”).

A sum score for the occurrence of any IPV victimization measure was computed by adding up the affirmative responses to the violent behaviors stated in the victimization measure of the CTS2S. In doing so, we created an indicator “IPV severity” for the *variety score* of the different assaultive behaviors or acts, 1 to 6 behaviors, by which one had been victimized. Participants with a higher sum score were victimized by a greater variability of violent behaviors than participants with a lower sum score. In this study, we therefore interpret our variety score of violent behaviors as a *severity measure* of IPV [60]. The question ‘Has […] happened?’ is a more accurate format than ‘How many times has […] happened?’ especially among respondents whose victimization experiences have lost their salience because they happen frequently. In addition, variety scores are less skewed than frequency scores. Therefore, they give equal weight to all violent acts [60]. Finally, previous research found the endorsement of more acts (i.e., a greater variety of violent acts) generally indicated greater severity as the most severe acts are least frequent [61].

*Change in IPV victimization during the pandemic*. Following each of the CTS2S items that had an affirmative response, an additional question was asked ‘How has this behavior changed during the pandemic?’ Response categories range from ‘less often’, ‘no change’, or ‘more often’, respectively. Each item indicates the perceived change in the individual respective experienced IPV behavior during the pandemic. Therefore, it is only possible to present changes in each individual IPV behavior during the COVID-19 pandemic, changes in CTS2S total score or subscale scores are not feasible.

### Statistical analysis

All analyses were computed with IBM SPSS statistics version 28 and stratified according to sex. Data were checked for completeness and attrition analyses were performed via Student’s t-tests and chi-square tests to detect any significant differences between completers and non-completers of the CTS2S in variables included in the current analyses (i.e., age, education, pregnancy status, being expectant first-time parent, household size, sex of partner, cohabitation, relationship satisfaction, dyadic coping, depression symptoms, anger-hostility symptoms, and social support). Additionally, data were analyzed descriptively to determine the prevalence of IPV victimization in women and men, and in the course of the revision process, chi-square was calculated to assess possible sex differences in the occurrence of any IPV victimization. Further, potential changes in IPV victimization during the pandemic in the affected sample was descriptively captured via percentages.

In the course of the revision process, we wanted to include the variables sex of partner and cohabitation as risk factors in our Pearson correlation and multiple logistic regression analyses. However, the vast majority of our sample were heterosexual (approx. 99%, see Table 1) and cohabiting (approx. 99%, see Table 1), which is why we excluded them from the analyses to prevent estimation problems due to empty cells in the joint distribution. We therefore computed Pearson correlation analyses to examine the associations of the following variables: occurrence of any psychological IPV victimization and age, education, pregnancy status, being expectant first-time parent, household size, relationship satisfaction, dyadic coping, depression symptoms, anger-hostility symptoms, and social support. The correlations between all risk factors were checked for multicollinearity, which was assumed with correlations *r* > .80 [62–64].

**Table 1:** Sample characteristics.

	Female (*n* = 468)			Male (*n* = 269)		
	*N* (%^a^)	*M*ean (*SD)*	Range	*N* (%^a^)	*Mean* (*SD)*	Range
**Age in years**	-	31.6 (3.9)	(20–43)	-	34.1 (4.9)	(24–55)
**Education**						
** ≤ 10 years**	66 (14.1)	-	-	41 (15.5)	-	-
** > 10 years**	402 (85.9)	-	-	224 (84.5)	-	-
**Pregnancy status**						
** Yes**	64 (13.8)	-	-	47 (17.6)	-	-
** No**	400 (86.2)	-	-	220 (82.4)	-	-
**Being expectant first-time parent**						
** Yes, being expectant first-time parent**	34 (7.3)	-	-	18 (6.7)	-	-
** No, already having one or more children**	430 (92.7)	-	-	249 (93.3)	-	-
**Household size**	-	3.2 (0.8)	(1–10)	-	3.1 (0.7)	(1–10)
**Sex of partner**						
** female**	2 (0.4)	-	-	258 (99.6)	-	-
** male**	456 (99.1)	-	-	1 (0.4)	-	-
**Cohabitation**						
** Yes, permanently**	453 (98.9)	-	-	255 (98.8)	-	-
** Yes, not permanently (e.g., only on weekends)**	5 (1.1)	-	-	3 (1.2)	-	-
** No**	0 (0)			0 (0)		
**Relationship satisfaction (PFB-K)**	-	19.6 (4.7)	(3–27)	-	19.2 (4.2)	(4–27)
**Dyadic coping (DCI)**	-	131.6 (19.4)	(62–175)	-	130.0 (17.7)	(78–169)
**Depression symptoms (EPDS)**	-	6.3 (4.3)	(0–22)	-	4.6 (4.3 )	(0–20)
**Anger–hostility symptoms (SCL-90-R)**	-	2.9 (3.3)	(0–22)	-	2.0 (2.6)	(0–16)
**Social support (F-SozU K-14)**	-	4.3 (0.7)	(1–5)	-	4.2 (0.6)	(2–5)

*Note*. All variables were measured at T1 DREAM_CORONA,_ except of *Education* which was assessed in the basic DREAM study. PFB-K = short version of the Partnership Questionnaire; DCI = The Dyadic Coping Inventory; EPDS = Edinburgh Postnatal Depression Scale; SCL-90-R = Subscale of the Symptom-Checklist Revised; F-SozU K-14 = short form of Fragebogen zur sozialen Unterstützung.

^a^ Valid percentage.

*N* varied between analyses due to missing values.

Next, we conducted a multiple logistic regression analysis to identify risk factors of IPV. The final model had the binary measure of occurrence of any IPV victimization as the outcome and age, education, pregnancy status, being expectant first-time parent, household size, relationship satisfaction, dyadic coping, depression symptoms, anger-hostility symptoms, and social support as risk factors. Results are given as adjusted Odds Ratios (aOR) with 95% confidence intervals (CI).

The regression models were calculated for women and men separately in order to detect differential risk factors of their IPV victimization. By doing this, we aimed to identify distinctions that may exist in the factors contributing to IPV for each sex. Finally, in the case of multicollinearity, we additionally conducted sensitivity analyses without variables that were highly correlated (i.e., depression and anger-hostility symptoms among women). The results remained consistent with the regression model including these variables, which is presented below. The statistical significance level was set at a p-value of 5%. Due to missing data in risk factors, *N* varied between analyses.

### Ethical considerations

The DREAM_CORONA_ study was approved by the Ethics Committee of the Technical University of Dresden (No: EK 278062015). Female and male participants were independently given written information about the aims and procedures of the study during recruitment. They were independently informed about pseudonymization of their data and their right to withdraw from the study at any time. All participants independently signed a declaration of written informed consent.

## Results

### Sample characteristics

The total sample consisted of 737 participants, with 64% female (*n* = 468) and 36% male (*n* = 269) participants. The mean (SD) age of the women was 31.6 years (3.9) and the age range was from 20 to 43 years. Only 13.8% (*n* = 64) were pregnant at the first assessment point (T1) of DREAM_CORONA_, with 7.3% (*n* = 34) being pregnant with their first child. For the female participants, 92.7% (*n* = 430) had one or more than one child. The mean (SD) age of men was 34.1 years (4.9) and the age range was from 24 to 55 years. Of them, 17.6% (*n* = 47) had pregnant partners, whereas 6.7% (*n* = 18) were expecting their first child. There were 93.3% (*n* = 249) of men who had one or more than one child.

The mean (SD) household size was 3.2 (0.8) for women and 3.1 (0.7) for men with a range of 1 to 10 people living in the same household for both female and male participants. The vast majority of female and male participants were in heterosexual relationships and lived with their partners permanently in one household. A summary of our sample’s characteristics can be found in Table 1.

### Attrition analyses

Attrition analyses were conducted for the included independent variables in the current analyses (i.e., age, education, pregnancy status, being expectant first-time parent, household size, sex of partner, cohabitation, relationship satisfaction, dyadic coping, depression symptoms, anger-hostility symptoms, and social support) for completers (i.e., participants who completed the IPV questionnaire at T2 of the DREAM_CORONA_ study) vs. non-completers. For women, non-completers had higher EPDS scores, meaning more depressive symptoms, than completers at T1 (*t* = 2.270, *p* = 0.024). For men, compared to completers, non-completers had less often pregnant partners (17.6% vs 9.3% *χ*^*2*^ = 4.727, *p* = 0.030) and had less often more than 10 years of school education (84.5% vs 75.2% *χ*^*2*^ = 5.104, *p* = 0.024). There were no differences between completers and non-completers regarding any remaining variable for women and men (tables on request).

### Prevalence of IPV victimization

Chi-square test results suggested that significantly more women than men experienced the occurrence of any IPV victimization (*χ*^*2*^ = 6.105, *p* = 0.013). Five in 10 women (n = 232, 49.6%) and 4 in 10 men (n = 108, 40.1%) faced at least one form of IPV in the last 12 months by their current partner (Table 2). Psychological aggression was the most prevalent form of IPV encountered by women and men, with 48.5% (n = 227) and 39.4% (n = 106), respectively. This was followed by physical assault with 2.6% (n = 12) and sexual coercion forms of violence (2.8%, n = 13) for women, as well as for men, with 3.3% (n = 9) and 1.5% (n = 4), respectively. Of those who experienced any occurrence of IPV in the last 12 months, 208 (89.7%) women and 97 (89.8%) men were victimized by a single violent behavior. One woman was found to experience a total of 5 out of 6 of the assessed abusive behaviors (Table 3).

**Table 2:** Prevalence of IPV victimization in the past 12 months.

	Female (*n* = 468)	Male (*n* = 269)
	*N* (%^a^)	*N* (%^a^)
**Occurrence of any IPV victimization**	232 (49.6)	108 (40.1)
**Occurrence of any psychological IPV victimization**	227 (48.5)	106 (39.4)
**Occurrence of any physical IPV victimization**	12 (2.6)	9 (3.3)
**Occurrence of any sexual IPV victimization**	13 (2.8)	4 (1.5)

Note. *Multiple answers possible*.

^a^ Valid percentage.

**Table 3:** IPV severity.

	Female (*n* = 232)	Male (*n* = 108)
	*N* (%^a^)	*N* (%^a^)
**One behavior**	208 (89.7)	97 (89.8)
**Two behaviors**	15 (6.5)	10 (9.3)
**Three behaviors**	8 (3.4)	1 (0.9)
**Four behaviors**	-	-
**Five behaviors**	1 (0.4)	-

*Note*. ^a^ Valid percentage.

### Change in IPV victimization during the pandemic

In Table 4, perceived change in *each* of the experienced IPV behaviors by women and men during the COVID-19 pandemic is described in total numbers and percentages. The changes were reported for each individual IPV behavior by women and men who were affected by their partner’s respective IPV behavior. The majority of women and men reported single behaviors of psychological and physical IPV victimization during the pandemic to have remained unchanged. Nevertheless, for certain IPV behaviors on the psychological and physical IPV victimization subscales, a portion of both affected women and men also reported higher frequencies during the COVID-19 pandemic (Table 4). Female and male participants who experienced sexual IPV victimization behaviors reported that these behaviors had not changed or were happening less often.

**Table 4:** Change in IPV victimization during the pandemic.

		Items	Less often *N* (%^a^)	No change *N* (%^a^)	More often *N* (%^a^)
**Affected female** **(*n* = 232)**	**Psychological aggression**	.. insulted or swore at me (*n* = 224)	10 (4.5)	153 (68.3)	61 (27.2)
		.. destroyed things, threatened (*n* = 7)	-	5 (71.4)	2 (28.6)
	**Physical assault**	.. pushed, shoved, slapped (*n* = 11)	-	8 (72.7)	3 (27.3)
		.. punched, kicked (*n* = 4)	-	3 (75.0)	1 (25.0)
	**Sexual coercion**	.. used physical force (*n* = 1)	-	1 (100)	-
		.. insisted, without using protection (*n* = 12)	1 (8.3)	11 (91.7)	-
**Affected male** **(*n* = 108)**	**Psychological aggression**	.. insulted or swore at me (*n* = 106)	2 (1.9)	79 (74.5)	25 (23.6)
		.. destroyed things, threatened (*n* = 2)	-	1 (50.0)	1 (50.0)
	**Physical assault**	.. pushed, shoved, slapped (*n* = 8)	1 (12.5)	6 (75.0)	1 (12.5)
		.. punched, kicked (*n* = 1)	1 (100)	-	-
	**Sexual coercion**	.. used physical force (*n* = 0)	-	-	-
		.. insisted, without using protection (*n* = 4)	2 (50.0)	2 (50.0)	-

*Note*. ^a^ Valid percentage.

N *varied between analyses due to missing values*.

### Risk factors of IPV victimization

Correlations between all variables are displayed in Table 5. Correlation analyses revealed significant associations between the occurrence of any IPV victimization and lower relationship satisfaction, lower dyadic coping, higher anger-hostility symptoms, and lower social support for female and male participants. They also revealed significant correlations between the occurrence of any IPV victimization and younger age, and higher depression symptoms for male participants only.

**Table 5:** Pearson correlation matrix.

	Variable	1	2	3	4	5	6	7	8	9	10	11
**Female**	**1. Occurrence of any IPV victimization**	1.00										
	**2. Age in years**	.010	1.00									
	**3. Education**	.082	.015	1.00								
	**4. Pregnancy status**	-.020	-.119*	.038	1.00							
	**5. Being expectant first-time parent**	.079	.156**	-.020	–.703**	1.00						
	**6. Household size**	.070	.311**	-.027	-.398**	.465**	1.00					
	**7. Relationship satisfaction**	-.359**	-.137**	-.032	.142**	-.247**	-.230**	1.00				
	**8. Dyadic coping**	-.279**	-.169**	.009	.163**	-.254**	-.197**	.721**	1.00			
	**9. Depression symptoms**	.055	-.011	-.001	-.080	.070	.072	-.166*	.194**	1.00		
	**10. Anger–hostility symptoms**	.204**	-.008	.003	-.125**	-.139**	.162**	-.341**	-.321**	.631**	1.00	
	**11. Social support**	-.180**	-.113*	.003	.036	-.114*	-.120*	.347**	.343**	-.196**	-.234**	1.00
**Male**	**1. Occurrence of any IPV victimization**	1.00										
	**2. Age in years**	-.158*	1.00									
	**3. Education**	-.013	-.119	1.00								
	**4. Pregnancy status**	.003	-.013	.087	1.00							
	**5. Being expectant first-time parent**	-.024	.036	-.075	-.582**	1.00						
	**6. Household size**	.037	.099	-.037	-.279**	.425**	1.00					
	**7. Relationship satisfaction**	-.307**	.009	-.067	.072	-.109	-.058	1.00				
	**8. Dyadic coping**	-.197**	-.085	-.058	.033	-.035	-.026	.717**	1.00			
	**9. Depression symptoms**	.130*	.079	.010	.098	.048	.069	-.225**	-.290**	1.00		
	**10. Anger–hostility symptoms**	.228**	.021	-.031	.050	.011	.100	-.153*	-.182**	.589**	1.00	
	**11. Social support**	-.138*	-.114	-.065	.025	.023	-.017	.430**	.519**	-.352**	-.232**	1.00

*Note*.

*p < .05

**p < .01

***p < .001.

According to our analysis of risk factors through the stratified multiple logistic regression model, we found that women’s higher relationship satisfaction was negatively associated with the occurrence of IPV victimization [aOR = 0.873; 95% CI: (0.81–⁠0.93)] and having higher symptom levels of anger-hostility was positively associated with the occurrence of any IPV victimization [aOR = 1.151; 95% CI: (1.04–⁠1.27)]. As for the male participants, we found that men’s younger age [aOR = 0.928; 95% CI: (0.87–⁠0.98)] and higher relationship satisfaction [aOR = 0.844; 95% CI: (0.75–⁠0.93)] were negatively associated with the occurrence of any IPV victimization. Similar to women, having higher symptom levels of anger-hostility was positively associated with the occurrence of any IPV victimization in men [aOR = 1.196; 95% CI: (1.03–⁠1.38)] (Table 6).

**Table 6:** Results of the multiple logistic regression models, with the occurrence of any IPV victimization as dependent variable.

Variables	Female (*n* = 412)		Male (*n* = 226)	
	aOR (95% CI)	P–Value	aOR (95% CI)	P–Value
**Age in years**	0.964 (0.91–1.02)	.207	**0.928 (0.87–0.98)**	**.021**
**Education**	1.294 (0.69–2.42)	.421	1.015 (0.42–2.41)	.974
**Pregnancy status**	1.783 (0.72–4.41)	.211	0.842 (0.31–2.24)	.731
**Being expectant first-time parent**	1.401 (0.41–4.68)	.584	0.452 (0.10–1.99)	.294
**Household size**	1.088 (0.78–1.51)	.616	1.177 (0.76–1.80)	.453
**Relationship satisfaction**	**0.873 (0.81–0.93)**	**< .001**	**0.844 (0.75–0.93)**	**.002**
**Dyadic coping**	0.995 (0.97–1.01)	.508	1.004 (0.97–1.03)	.756
**Depression symptoms**	0.946 (0.88–1.01)	.105	0.957 (0.87–1.04)	.341
**Anger–hostility symptoms**	**1.151 (1.04–1.27)**	**.006**	**1.196 (1.03–1.38)**	**.015**
**Social support**	0.816 (0.57–1.15)	.252	0.961 (0.52–1.75)	.756

*Note*. *N* varied between analyses due to missing values.

Further, when we conducted sensitivity analyses by removing the highly correlated variables identified (e.g., *r* = .631, *p* < .001 for depression and anger-hostility symptoms among women), we found that the results remained consistent with our initial findings from the original multiple logistic regression. This suggests that the observed relationships and effects are robust and not heavily influenced by the presence of multicollinearity (tables on request).

## Discussion

This research focused on shedding light on the understudied issue of female and male victimization of perinatal IPV in Germany. The aim of our study was to examine the prevalence of psychological, physical, and sexual IPV victimization in (expectant) mothers and fathers. Further, the study examined potential changes in any of the experienced violent behaviors during the unprecedented circumstances of the COVID-19 pandemic. Finally, our study sought to explore potential risk factors of perinatal IPV victimization.

### Prevalence of IPV victimization in the last 12 months

Our findings revealed that 49.6% of women and 40.1% of men faced at least one occurrence of any IPV victimization in the last 12 months by their current partner. Our physical and sexual 12-month IPV rates were similar to pre-pandemic estimates in Germany [65]. There, physical and/or sexual IPV 12-month prevalence estimates accounted for 3% for women by their current partner [65]. One of the most frequent forms of IPV in western societies is psychological IPV [66], which involves behaviors intended to generate emotional harm or threat of harm, such as belittling, humiliating, threatening, or intimidating the victim, and it also appeared to be the most prevalent form in women and men in our study. This corresponds to the latest EU-wide survey, where 12-month psychological IPV by a current and/or previous partner was found at 50% for women [65], as well as with other studies conducted in the USA [67] and South Africa [68], which included only female participants.

Given that the vast majority of victimized women and men experienced at least some of these behaviors here, careful future research is warranted, as these behaviors may, among other things, have deleterious consequences for the quality and stability of relationships. Our IPV severity measure garnered empirical evidence that psychological abuse was in many cases not accompanied by physical or sexual violence. However, despite the detrimental impact of psychological abuse on victims, such a form of violent behavior is often difficult to prosecute as an abusive one [69–71]. More so, the strong link between psychological and physical IPV has previously been established, where psychological violence often precedes physical IPV, and it is considered one of its main risk factors [72]. Further, those who experience psychological aggression without physical aggression may not recognize such aggression, leading to prolonged exposure and negative consequences, such as PTSD in male and female victims [73,74].

Legislative reforms and laws are needed to support those affected by the health and economic consequences of violence, including psychological, physical, and/or sexual IPV, which can have several significant benefits in the lives of those affected. In Germany, it is important to note that while reforms, such as the *“Neues Soziales Entschädigungsrecht”[social compensation law] under the law SGB XIV*, can provide valuable benefits, they should ideally be part of a comprehensive approach to addressing IPV, especially now that the reformed law will provide compensation to victims of psychological violence starting from January 2024 [75]. The compensation can include quick and unbureaucratic access to psychotherapeutic services, medical and nursing services, welfare and participation services with the aim to offset the health and economic consequences suffered by the affected. In implementing such reform and in the case of IPV, it may be crucial to accompany the implementation with an evaluation of the possible outcomes through empirical research, where the forms, causes, and consequences of IPV for both women and men can be better understood in the light of what this reform might bring [76].

### Change in IPV victimization during the pandemic

Recent research emphasized the necessity of directing additional scientific attention to changes of IPV victimization during the COVID-19 pandemic [18]. Indeed, changes in each of the experienced behaviors during the pandemic varied in our sample. The majority of affected female and male participants reported no change in their reports of certain IPV behaviors on the psychological and physical IPV victimization subscales, meaning that their experiences of IPV had neither increased nor decreased and their affectedness had remained the same. However, of the victimized women and men, some reported an increase in experiencing them. The increase was also found in other empirical studies conducted in Iraq [77], India [78], Peru [79], Ethiopia [80], Tunisia [81], which included only female participants, as well as in Germany, where it was also found in men [19]. This increase in IPV victimization seen in our sample may reflect a group of IPV victims whose experiences of violence could be a function of the temporary isolation resulting from social distancing measures and stay-at-home orders imposed as a negative impact of the pandemic [33] when the global lockdowns were first enforced [82].

Furthermore, this increase in IPV victimization seemingly contradicts previous reports of administrative data in Germany, where a decrease in police reports had been documented from the beginning of March 2020 [83,84]. This seemingly contradiction might in fact reflect a possible alteration in help-seeking behaviors, as well as barriers to accessing services caused by policies related to the COVID-19 pandemic [85]. With this in mind, future studies could also look at possible post-pandemic changes in help-seeking behaviors. Our findings of no change in the level of victimization reported by most affected men and women indicate that they continue to experience IPV at the hands of their current partner. It also suggests a continuing need for support services. Public awareness may have been needed to improve access to these services by adapting them to the realities of the pandemic. This may also require sustained long-term action in the post-pandemic period.

### Risk factors of IPV victimization

In our stratified multiple logistic regression models, higher relationship satisfaction in women and men was negatively associated with the occurrence of any IPV victimization, which was also found in a previous study [86]. On the one hand, it highlights the potential protective role of healthy relationships in reducing the risk of interpersonal violence. On the other hand, one could also assume that higher relationship satisfaction could be an expression of being in a relationship, where violent behaviors are not practiced.

Higher levels of anger-hostility symptoms were positively associated with the occurrence of any IPV victimization for both women and men. This may indicate that individuals with elevated anger-hostility symptoms are more likely to experience IPV. Adjusting to the psychological burden, which might result from dealing with external stressors relating to the pandemic, could be a possible reason for having higher levels of symptoms in the first place [32,87]. Having higher anger-hostility symptoms could also be an expression to an already difficult relationship, where IPV could be pre-existing and/or bidirectional.

In summary, our multiple logistic regression results provide valuable insights into the significance of relationship satisfaction and anger-hostility symptoms for both (expectant) women and men in understanding and addressing IPV. Although significantly correlated in the bivariate analyses, lower dyadic coping and social support were not associated with the occurrence of any IPV victimization for perinatal women and men in our multiple regression models, nor was higher depression for perinatal men.

### Strengths and limitations

The current study has several strengths and limitations. First, our IPV prevalence analysis and regression models covered nearly the complete first year of COVID-19 in Germany. Second, recruitment for this study was completed in 2020, providing a snapshot of the magnitude of and factors associated with IPV during a critical period marked by the onset of the COVID-19 pandemic. Third, our inclusion of perinatal men was of great importance, as a previous study showed that men who seek help or services for IPV victimization feel that their concerns are not taken seriously [88]. A qualitative study by Brooks et al. (2020) further highlighted that some men expressed fears of skepticism and lack of support when disclosing their experiences of IPV [89]. These reactions have profound implications, causing individuals to doubt their own perceptions and experiences, thereby exacerbating trauma or other psychological symptoms. With this in mind, it was important to explore (expectant) men’s victimization in our study and the relationship of the different factors to it, in order to provide insights into their experiences and a starting point for addressing them. Fourth, our data are based on the actual experience of our participants and are therefore not biased by help-seeking behaviors, which might have been altered by the pandemic. Fifth, knowledge of risk factors can be crucial from a prevention perspective, as it provides insight into factors that could prevent or reduce the occurrence of any form of IPV. Nevertheless, when interpreting the results, one should keep in mind the limitations of this study.

First, our analysis of changes in exposure to experienced IPV behaviors during the pandemic was limited by the retrospective assessment. Due to the nature of our data collection, our assessment of changes was carried out for each of the experienced IPV behaviors and we were only able to present our results descriptively. Second, IPV victimization was assessed for the prior 12 months at T2, whereas the T2 measurements were conducted already 5 months after the T1 measurements. Thus, we acknowledge the infeasibility of drawing causal conclusions regarding the relationship between IPV victimization report and the explored risk factors. Furthermore, our exploration and interpretation of the results were conducted on an individual basis rather than through a dyadic lens. This approach entails certain limitations, confining our findings to self-reported individual experiences and failing to capture the full constellation of dynamics that define the partnership. As a result, our ability to synthesize a comprehensive and accurate picture of the context in which the violence occurred is limited. Even though there was a lack of interdependence within our sample, this approach allowed us to examine perinatal IPV victimization faced by each sex independently. Third, our population-based sample contained mostly well-educated and relatively young (expectant) parents with singleton pregnancies. Our findings can therefore not be generalized to other populations such as (expectant) individuals of twins or multiples, older individuals, or to at-risk groups, such as sexual and gender minorities, who may be disproportionately affected by pandemic-related stressors relating to employment, finances, and psychological health [90,91].

### Future research directions

Given the limitations of our study, and to overcome the retrospective nature of our analysis, future studies should use a prospective design with regular assessments. Since psychological IPV was found to be most prevalent in our study, we recommend future studies to include measures that could better assess it, where behaviors relating to coercive control (e.g., restricting a partner’s movements, jealousy and suspicion, use of children in the forms of, for example, neglecting their needs or threatening to kidnap them) [92,93], and economic violence (e.g., financial control) could be included. This in turn could enhance our understanding of the role of psychological violence in intimate relationships and its consequences on family life.

In the case of our exploratory analysis of the changes in IPV victimization during the COVID-19 pandemic, we highlight the development of comprehensive indices that capture the changes in the forms of IPV, rather than exploring the change for individual IPV behaviors. Because repeated assessments of the CTS2S to measure changes in IPV victimization is only feasible if there is a gap of more than one year between measurement points, as the CTS2S collects data for the previous 12 months, an alternative comprehensive measure or index is needed. Future research should consider constructing such indices within the affected population, which could allow for a nuanced examination of changes in IPV and its forms during societal crises such as the COVID-19 pandemic.

Our findings also highlight the need for greater examination of and support for individuals who reported no change in psychological, physical, and/or sexual IPV victimization behaviors, and individuals who reported an increase in psychological and physical IPV victimization behaviors during the pandemic–groups of particular importance who may be vulnerable to continued and/or increased IPV in the future. This emphasizes the urgency of exploring the underlying factors that contribute to the risk of IPV among those who continued to experience IPV and who experienced an increase, particularly during the perinatal period. This finding is consistent with existing research highlighting the perinatal period as a time of vulnerability and multiple health consequences [7–12].

Moving beyond individual analyses, future research should adopt a dyadic lens to examine IPV in the context of intimate relationships. For instance, when looking into the factor relationship satisfaction, future dyadic studies could be conducted, examining the interdependent dynamics in relationships [94,95]. In this way, a truer image of the context of violence in intimate relationships could be provided.

Further, future studies should intentionally recruit participants from under researched populations such as men in hetero relationships, and at-risk populations such as sexual and gender minorities. Finally, to enhance the preventive perspective, future research should explore additional risk factors not covered in our study. These could include specific stressors that could be relevant in the context of societal crises such as employment uncertainties and financial strain, especially within at-risk populations.

## Conclusions

In the current study, psychological IPV victimization was present in almost every second (expectant) couple. The majority of affected women and men disclosed no change in their IPV victimization, suggesting that they continued to experience psychological and physical IPV during the pandemic. Some reported an increase in psychological and physical IPV victimization, which supports the previously documented increase in IPV for female participants during the pandemic.

While our study provides valuable insights into the early stages of the pandemic, it highlights the importance of continued research into the temporal dynamics of IPV. This line of research will not only enhance our understanding of the potential long-term consequences, but will also contribute to the development of evidence-based interventions that can mitigate the impact of prolonged crises on intimate partner relationships.

In addition, the findings from this study could highlight the need to develop gender-sensitive strategies that take into account different vulnerabilities. For instance, the inclusion of the other partner in IPV research could provide more context to better understand and prevent IPV in samples that include both (expectant) partners. Because perinatal IPV victimization requires appropriate support and resources for those who are most significantly affected by it, including men, accurate measurement of differences in IPV experiences between partners is essential. And in order to obtain ’accurate’ results of IPV, experiences of violence need to be measured in ways that could capture the context of violence (e.g., examining severity, patterns, and coercive control). In addition, future research could examine the dyadic dynamics and the interdependent interactions between partners to provide a more comprehensive picture of IPV. This could provide contextual understanding to guide interventions and policies aimed at reducing the prevalence and risk of perinatal IPV.

Ultimately, the findings of this study have the potential to bring about meaningful change by guiding the development of targeted strategies to prevent and respond to IPV. As a concrete example, research initiatives should be aligned with the German government’s initiatives to support victims of violence, including those affected by psychological IPV, as highlighted by recent policy reforms. By recognizing and addressing the challenges faced by male victims in perinatal IPV scenarios, our research not only fills critical gaps in existing knowledge, but also aligns with the broader societal goal of promoting a safer and more supportive environment for all individuals affected by IPV.
